# Treatment of Sewage Using a Constructed Soil Rapid Infiltration System Combined with Pre-Denitrification

**DOI:** 10.3390/ijerph15092005

**Published:** 2018-09-14

**Authors:** Wenlai Xu, Jinyao Chen, Yue Jian, Zhicheng Pan, Zishen Mou

**Affiliations:** 1 State Key Laboratory of Geohazard Prevention and Geoenvironment Protection, Chengdu University of Technology, Chengdu 610059, China; xuwenlai1983@163.com (W.X.); txgsfy@163.com (J.C.); 2 Haitian Water Group Stock Company, Chengdu 610059, China; pan12487616@126.com; 3 Department of Chemical and Engineering, Tokyo University of Agriculture & Technology, Tokyo 1848588, Japan; 4 Environmental Research Institute, ChongQing Academy of Animal Sciences, Chongqing 404100, China; nstxdy@163.com

**Keywords:** COD, constructed soil rapid infiltration system, nitrogen, pre-denitrification, wastewater treatment

## Abstract

The activated sludge process of the anaerobic/oxic (A/O) process has a good denitrification performance because it can make full use of the carbon source in the original sewage, and the denitrification can provide alkalinity for aerobic nitrification. The traditional constructed soil rapid infiltration (CSRI) system, on the other hand, has a poor nitrogen removal effect. Dividing the traditional CSRI system into two sections, one performs denitrification as an anoxic section, while the other performs nitrification as an aerobic section and is placed after the anoxic section. The nitrification liquid of the effluent from the aerobic section is mixed with the original wastewater and enters the anoxic section for denitrification. We expected that this would be improved by combining CSRI with a pre-denitrification step that would make full use of the carbon source in the original sewage. In a small-scale experimental model, the removal efficiencies of nitrogen, in the form of ammonium, nitrate, and total nitrogen (TN), as well as chemical oxygen demand (COD), were determined. The hydraulic load was varied, while the backflow reflux capacity was kept constant, to determine the effect on the pre-denitrification process. An average removal rate of 95.4% for NH_4_^+^-N and 96% for COD could be obtained when a hydraulic load of 80 cm^3^(cm^2^·d)^−1^ and a reflux ratio of 75% were applied. Under these conditions, the average removal rate of TN was 77.4%, which is much higher than what can be typically achieved with conventional CSRI systems.

## 1. Introduction

With increasing pressure on water sources, a highly efficient and low-cost sewage treatment technology is urgently needed. Anammox, short-cut nitrification and denitrification, ion exchange, and wetlands have a good effect on denitrification and are currently the subject of significant research and development [1,2]. In this paper, the constructed soil rapid infiltration (CSRI) system, a new sewage biofilm treatment technology, is studied. CSRI is a new sewage ecological treatment method established by Professor Zhong Zuoshen of the China University of Geosciences based on a rapid infiltration system (RI), which makes up for the disadvantages of the low hydraulic load rate of constructed wetlands. It consists of a screen pool, pre-sedimentation pool, rapid infiltration pool, and water outlet system. Artificial filter materials, such as river sand, zeolite, and iron powder, fill the rapid infiltration pool, and the operation mode adopts wet-dry cycling for sewage treatment. The period of the hydraulic load is short, meaning frequent flooding and frequent drying, and the hydraulic load is 1.0–1.5 m/d [3]. CSRI absorbs, intercepts, and decomposes pollutants in sewage wastewater by percolating media and microorganisms on the media. Its unique structure and water intake mode of wet–dry cycling make the microorganism phase on the surface of the percolation media very rich. The percolation medium has aerobic, aerobic-aerobic, and anaerobic functions, so CSRI has a good effect on wastewater treatment. The flooding period, that is, the water distribution period, is mainly for the sake of allowing the filters to adsorb and intercept pollutants in the sewage. During the drying period, that is, the period after the water distribution has finished until the next water distribution period, the microorganisms on the sand and gravel transform the adsorbed pollutant to prevent sand and gravel adsorption saturation and maintain the pollutant adsorption capacity of the filter. In relation to the treatment of municipal wastewater, the CSRI system is characterized by low installation costs, a low operative energy consumption, ease of operation, and a sufficient quality of the produced effluent. The CSRI system has been applied in China and other countries, but it suffers from a low efficiency in removing nitrogen [3,4,5], which currently limits any further application and promotion. There are two reasons for the low nitrogen removal efficiency of traditional CSRI: First, a large number of ammonia nitrogen and organic carbon sources in one water source are trapped in the upper layer of the filter tank, with sufficient oxygen in the upper layer of the filter material. This environment is favorable for nitrifying bacteria, but restricts the growth of denitrifying bacteria. Therefore, ammonia nitrogen is converted into nitrate nitrogen under the action of nitrifying bacteria, which limits denitrification. Then, less air reaches the lower filter layer, which is suitable for the growth of denitrifying bacteria. However, denitrifying bacteria are heterotrophic microorganisms, so sufficient carbon sources are needed. Most carbon sources in sewage are absorbed in the upper layer of the filter, and the organic carbon sources in the lower layer are limited, which limits the growth of denitrifying bacteria. In general, CSRI does not provide a good environment for denitrifying bacteria and thus inhibits denitrification. It is imperative that the removal of total nitrogen (TN) should be improved.

Processing activated sludge by an anaerobic/oxic (A/O) process has a satisfactory denitrification performance because it can make full use of the carbon source in the original sewage, while denitrification provides the alkalinity required for aerobic nitrification. This has been widely applied in a variety of processes. For instance, Tan et al. [6] combined pre-denitrification with a submerged membrane bioreactor to treat domestic sewage, and discussed the effect of internal circulation and aeration on the system’s operation. Another study treated sewage with a low COD/TN ratio by combining pre-denitrification with an anaerobic/anoxic/aerobic sequence batch reactor [7]. The combined removal of phthalic acid diethyl ester and nitrogen from sewage by pre-denitrification in combination with biological filters has also been demonstrated.

Here, we combined pre-denitrification with CSRI and applied this to the treatment of synthetic sewage in a model system, in which the process was cycled by circulation to achieve sufficient purification. The removal of chemical oxygen demand (COD) and nitrogen in the form of ammonium (NH_4_^+^), nitrate, and TN was measured over time and under various hydraulic loads, while the amount of circulation cycling was kept constant. We aimed to identify the optimal working condition parameters in a model system to provide a theoretical basis for the denitrification treatment of domestic sewage by pre-denitrification in a CSRI system.

## 2. Materials and Methods

### 2.1. Experimental Sewage

The sewage used in this experiment was a mixture of artificial sewage and real domestic sewage at a ratio of 30:1. The COD content varied between 118.3 and 150.2 mg L^−1^, with an average of 129.6 mg L^−1^; nitrogen in the form of ammonium (NH_4_^+^–N) varied from 30.9 to 41.2 mg L^−1^ (average 36.7 mg L^−1^); nitrate nitrogen (NO_3_^−^–N) varied from 1.2 to 0.1 mg L^−1^ (average 0.7 mg L^−1^); total nitrogen TN varied from 38.9 to 45.2 mg L^−1^ (average 40.4 mg L^−1^); and the pH varied from 6.61 to 6.68, with an average of 6.72. The physicochemical characteristics of the sewage were determined and are shown in Table 1. The crucial parameters of this mixture were determined by the following methods, and these varied over time due to variation in the domestic sewage: NH_4_^+^-N was tested by Nessler’s Reagents spectrophotometer method; TN: Alkaline Potassium persulphate—UV spectrophotometry method; NO_3_–N: UV spectrophotometry method; and COD: Potassium dichromate digestion method. The analysis method is based on the fourth edition of the water and wastewater monitoring and analysis method [8].

### 2.2. Data Analysis

All data were conducted in triplicate and expressed as mean ± standard deviation (SD). An analysis of variance was executed to compare and validate the mean values measured. The removal rate of COD, NH_4_^+^-N, and TN was determined according to the formula:

*I* = (*C_in_* − *C_out_*) × *C_in_*^−1^ × 100%

where *I* is the removal rate, and *C_in_* and *C_out_* are the concentrations of the influent and effluent of each stage of CSRI, respectively.

### 2.3. Experimental Setup and Operation Mode

**Figure 1:**
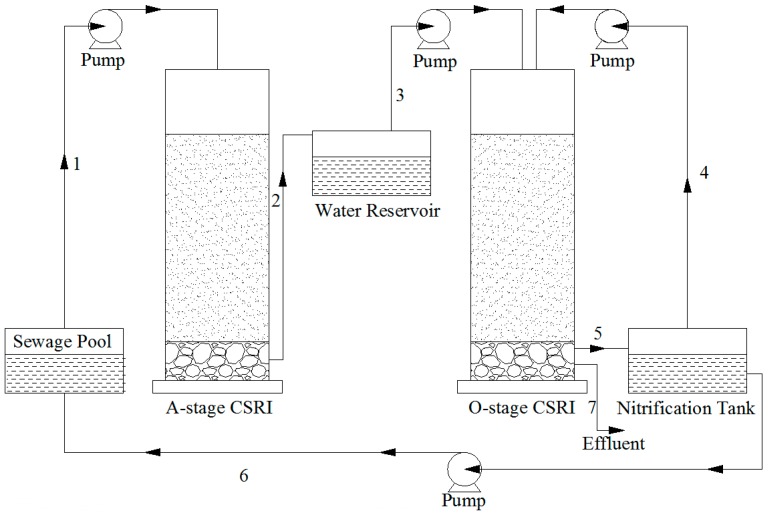
Schematic of the experimental constructed soil rapid infiltration (CSRI) model. For an explanation of the routes numbered 1 to 7, see text. The figure is not drawn to scale.

The experimental CSRI model is shown schematically in Figure 1.

The whole experimental system consists of an anaerobic (A) denitrification stage and an aerobic (O) nitrification stage, which take place in separate columns, while a major part of the water flow is circulating, and are followed by a nitrification reservoir. Carbon removal takes place during both the A and O stages because of microbial activity. The CSRI columns are made of Plexiglas tanks, with a column height of 70 cm and an inner diameter of 10 cm. Both tanks contain a 50 cm high filtration medium, laid on top of a 10 cm high pebble support layer. The filtration medium of tank A consists of 90% river sand and 10% zeolite sand, while that of tank O contains a mixture of 90% river sand, 5% zeolite sand, and 5% marble sand. The water inlet is at the top of both tanks, with a constant water table for A, due to a raised outlet that reduces oxygen as required for anaerobic denitrification. In contrast, by means of a water reservoir in between A and O, a drain/flood regime was applied to tank O to ensure oxygenation. The outlets of both tanks are located on the support layer, as depicted in Figure 1.

During operation, artificial sewage water is allowed into the sewage pool four times daily, and 20 cm^3^/cm^2^ of sewage is allowed into the system each time. The A-stage operates with a constant hydraulic load of 20 cm^3^/cm^2^ (arrow 1 in Figure 1) and a constant outlet of the same capacity each time. The water reservoir that captures the sewage leaving tank A (arrow 2) is emptied into tank O (arrow 3) by an infusion valve that opens during distribution. This completely floods the filtrate of tank O, which is completely emptied into the nitrification tank by opening outlet 5. Of the effluent collected in the nitrification reservoir, 15 cm^3^/cm^2^ (by default 75%) is fed back into tank A (arrow 6) as a continuous reflux. When outlet 5 is closed, outlet 7 can be opened to discharge the effluent, but outlet 7 must be opened after outlet 5 is closed. Moreover, after 2 h, when the sewage has been distributed, 5 cm^3^/cm^2^ of nitrification in the reservoir is fed back into tank O (arrow 4) to enhance reoxygenation. The whole system’s sewage disposal time is automatically controlled by the switch. After the system was steadily running, samples were taken every 48 h in accordance with the process. The equivalent of 80 cm^3^ (cm^2^·d)^-1^ and 60 cm^3^ (cm^2^·d)^−1^ (a reflux ratio of 75%) were applied during the reflow and were calculated on a daily basis.

## 3. Results and Discussion

### 3.1. Optimizing the Rate of Reflux

**Figure 2:**
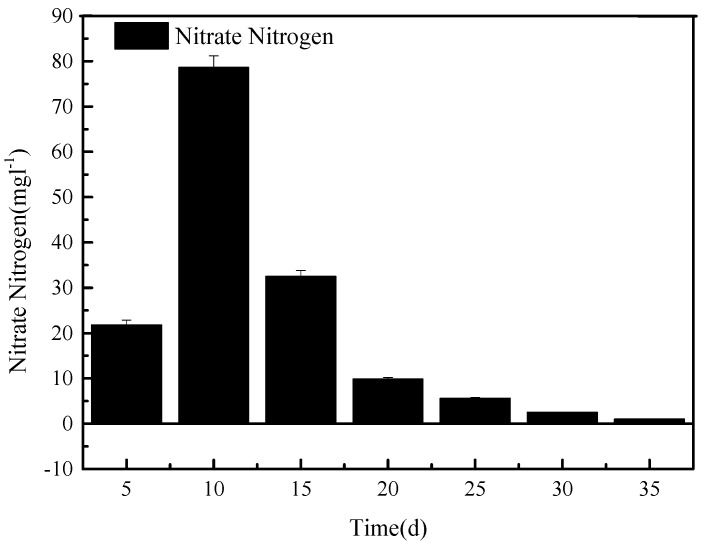
The concentration of nitrate determined at outlet 5 over 35 min of initial operation. The first sample was taken 5 min after outlet 5 was opened.

After the system was set up, sewage was loaded into tank A, and the initial flood/drain round of column O was sampled at outlet 5 every 5 min to investigate the initial denitrification process over a period of 30 min. During this experiment, outlet 7 remained closed, and outlet 5 was open. The nitrate content of the samples taken at outlet 5 is shown in Figure 2.

As can be seen in Figure 2, between 5 and 10 min after opening outlet 5, the content of nitrate in the effluent of tank O increased and then decreased after 10 min. After a flow of 20 min from outlet 5, the nitrate concentration in the sewage was less than 10 mg L^−1^, after which it only slightly decreased for the remaining 15 min. Therefore, for further experiments, the opening time of outlet 5 for sewage entering the nitrification reservoir was set to 20 min.

The temporary raise in the nitrate content of the sewage as it left tank O can be explained by the high initial nitrate content of the original sewage. Nitrate is highly soluble in water and, being negatively charged, will not adsorb well on the negatively charged filtration medium in tank O. Instead, it seems to be washed out during the first 15 min. Most of the nitrate nitrogen was concentrated in the first 20 cm^3^/cm^2^ of the water and was discharged into the nitrification tank.

### 3.2. The Removal Efficiency of COD

Following pilot experiments (data not shown), for the next experiment, the circulation flow (arrow 6) was set at 60 cm^3^ (cm^2^·d)^−1^, corresponding to 75% of the total water flow. After 2 h, when the sewage had been distributed, 5 cm^3^/cm^2^ of nitrification in the reservoir was fed back into tank O (arrow 4) to enhance reoxygenation. Outlet 7, which is discharged directly into the environment, was opened only after outlet 5 was closed. After loading and running the system for 48 h, samples were taken every 48 h for 44 days.

**Figure 3:**
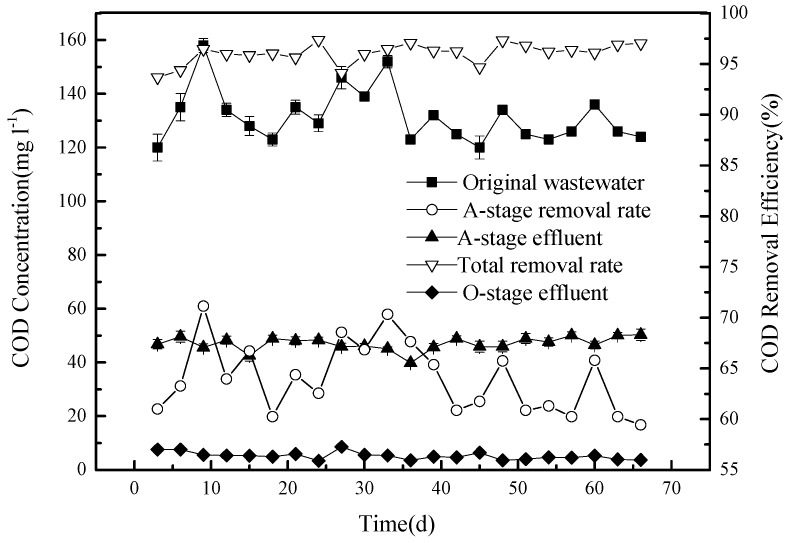
The concentration of COD (black symbols, left axis) at various stages of the system, and the removal efficiency in % of COD (open symbols, right axis).

The amount of COD varied over time in the original domestic waste water, resulting in variation of the sewage at the inlet, which is represented by the black squares in Figure 3.

This concentration (on average, 129.6 mg L^−1^) was reduced, following stage A, to around 50 mg L^−1^ (black triangles pointing upwards), producing a removal efficiency of COD of around 60% (white triangles pointing up). There are several reasons for the removal of COD at this stage: (1) The anaerobic tank A contains large numbers of denitrifying bacteria. The presence of both nitrogen, in the form of nitrate, and a rich carbon source supports their metabolic activity, consuming carbon during the process; (2) since the COD in the circulation fluid is lower, circulation dilutes the concentration of the original sewage in tank A; and (3) the filtration medium may adsorb some organic matter, removing COD from the liquid phase.

The COD concentrations, determined at outlets 5 and 7 of the O tank, are very similar, with concentrations below 10 mg L^−1^ (black triangles pointing down in Figure 3), producing an overall removal rate of COD of 93% or more compared to the original waste water (white triangles pointing down). Thus, while stage A, in this model of CSRI combined with pre-denitrification, removes a large amount of COD from the effluent and considerably reduces the COD concentration, the COD is further reduced at the O stage, in part by adsorption, and is retained in the filtering medium. Microorganisms present in the O tank can also degrade organic matter. With sufficient oxygen present, biodegradable organic macromolecules would first be decomposed into smaller molecules by enzymes produced by the microorganisms and then be fully degraded. The organic substrates would be used for growth and would also be transformed into CO_2_, CO_3_^2−^, H_2_O, NO^3−^, SO_4_^2−^, and PO_4_^3−^. System O runs with a dry phase that lasts six times longer than the wet phase, and it is watered after each sewage distribution of 2 h with 5 cm^3^ (cm^2^)^−1^ to enhance the reoxygenation effect of the O-stage system. It has been shown that this also facilitates the decomposition of organic matter in the system [9,10,11,12].

### 3.3. The Removal Efficiency of NH_4_^+^-N

**Figure 4:**
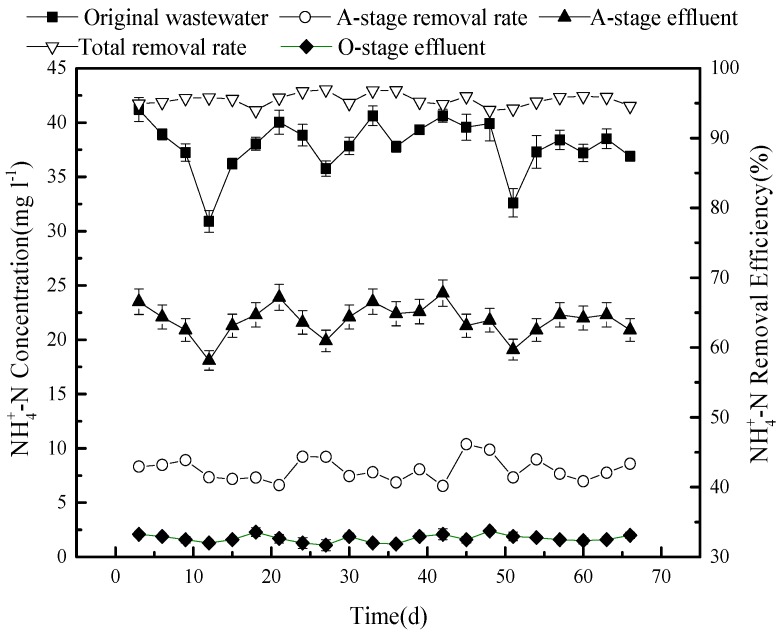
The concentration and removal efficiency of NH_4_^+^-N in the system.

The removal efficiency of NH_4_^+^-N, tested during the same experiment, is shown in Figure 4.

The concentration of NH_4_^+^-N in the sewage was, on average, 36.7 mg L^−1^ (30.9–41.2 mg L^−1^), and this was reduced to 18.1–24.3 mg L^−1^ in the effluent of stage A, producing a removal efficiency of approximately 40%. The observed removal dynamics is the result of a combination of factors: (1) The main factor is probably the dilution of the mixed circulation solution with the original sewage; (2) since the reflux solution and the original sewage may be mixed with a certain amount of dissolved oxygen, the NH_4_^+^-N conversion may at first be limited; (3) however, after the dissolved oxygen in the sewage is used up, an anaerobic environment is formed, allowing anaerobic ammonium oxidation during stage A; and (4) some NH_4_^+^-N will be adsorbed by the solid matter. The latter is quite obvious at the beginning of the operation. As adsorption sites become saturated, the adsorption becomes weaker, after which the adsorption and resolution are balanced [13,14,15]. The effects of adsorption and anaerobic ammonium oxidation during stage A are probably lower than the dilution effect of the reflux nitrification solution.

The concentration of NH_4_^+^-N at outlets 5 and 7 are comparable in the range of 5 mg L^−1^ or less. This brings the overall removal rate of NH_4_^+^-N in this model CSRI system to at least 93%. This high efficiency can be obtained because the O-stage is operated by regular flooding and draining. When flooded, the NH_4_^+^-N in the liquid is adsorbed and retained by the filtering medium, after which it can be converted into nitrate or nitrite by autotrophic aerobic nitrifying bacteria. The process depends both on the presence of oxygen and suitable carbon sources. The efficiency can thus be adjusted by varying the timing and duration of the dry and wet phases, while in a scaled-up model, plant roots would produce ventilation ducts to further improve reoxygenation. When a wet phase, with a flow of 5 cm^3^ (cm^2^)^−1^, was applied after the sewage had been distributed, 2 h of nitrification took place [16,17,18]. A highly concentrated carbon source would support the growth and metabolism of nitrifying bacteria, which would easily outcompete heterotrophic bacteria.

Because of the pre-denitrification in this system, denitrification will consume a certain amount of carbon during stage A. The reflux solution has a lower concentration of carbon, which further dilutes the carbon source of the original sewage. The limited concentration of carbon in the O-stage system increases the competitiveness of nitrifying bacteria. At the same time, the denitrification process also provides a certain amount of alkalinity for the nitrification section.

The following nitrification reactions take place in stage O:

2NH_4_^+^ + 3 O_2_ → 2NO_2_^–^ + 4H^+^ + 2H_2_O (1)

2NO_2_^–^ + O_2_ → 2NO_3_^–^ (2)

Denitrification (with the substrate methanol as an example), mainly in stage A, would take place as:

2NO_3_^–^ + 5 CH_3_OH → 5CO_2_ + N_2_↑ + 7H_2_O + 6OH^–^ (3)

From this example, it can be seen that with an oxidation of 2 mol NH_4_^+^, 4 mol H^+^ is produced, and when 2 mol NO_3_^–^ is reduced, 2 mol OH^–^ is produced. The sewage leaving stage A will increase the pH (increasing alkalinity) in stage O, which can reduce the supply of alkalinity in the O-stage of the CSRI system. 

### 3.4. The Removal Effect of Nitrate and Total Nitrogen

**Figure 5:**
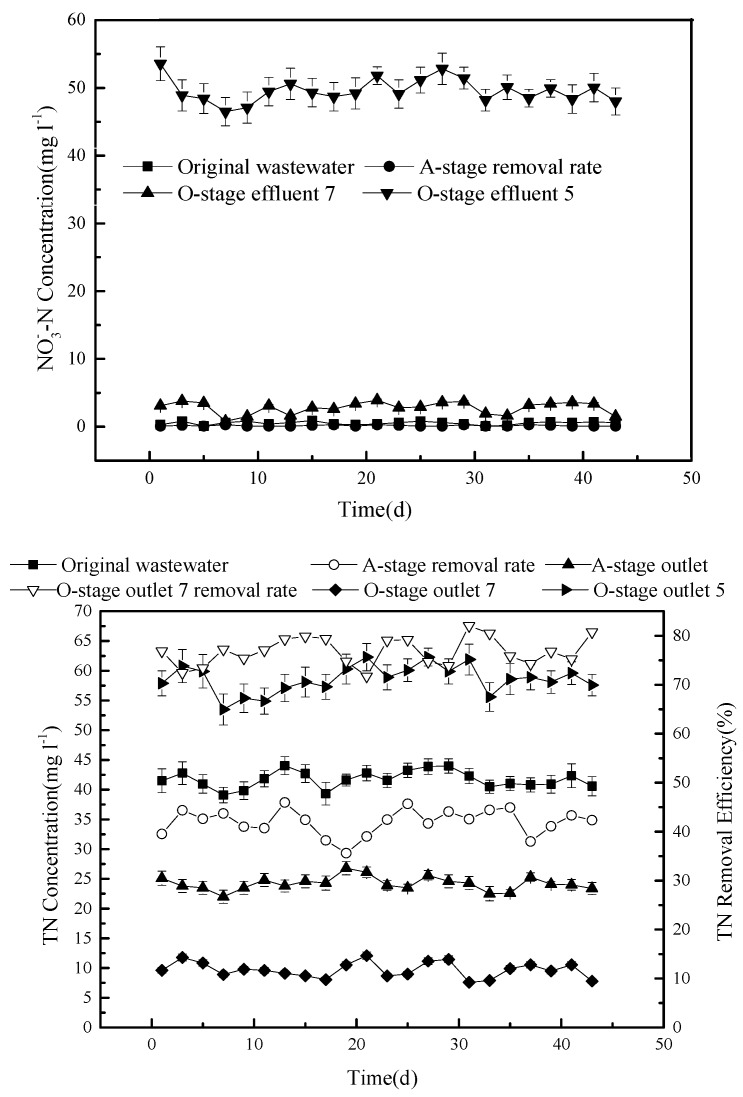
The changes of nitrate concentration (**top**) and of total nitrogen (TN) (**bottom**) during the process.

The concentration of nitrate during the experiment was also determined (Figure 5).

The concentration of the NO_3_^–^ discharged from outlet 5 is relatively high, with an average of 49.2 mg L^−1^, while it is very low in the effluent of stage A (0.52 mg L^−1^) and only slightly higher than that in the original sewage (0.16 mg L^−1^). Outlet 7 produces an average concentration of 3.8 mg L^−1^, which is more than double that in the original sewage.

Overall, the nitrate concentration changes follow the same trend as that of the total nitrogen (Figure 5). The average concentration of TN in the original sewage (41.7 mg L^−1^) is reduced to around 24 mg L^−1^ in the effluent of stage A, giving an average removal efficiency of about 42%. The average TN concentration in the effluent, discharged from outlet 7 after stage O, is reduced to about 9.6 mg L^−1^, producing an average total removal efficiency of 76.7%. When measured from outlet 5, the TN concentration is 58.5 mg L^−1^, which is actually higher than that in the original sewage.

As shown in Figure 2, part of the effluent flows back into the system, whereby a large amount of nitrate, produced in the nitrification reaction, is dissolved. As the process continues, the nitrate concentration decreases. The filtering medium can also adsorb a limited amount of nitrogen in the form of NH_4_^+^. For these reasons, the TN concentration measured at the outlet 5 can be high, while it is low at outlet 7. The nitrification solution carries a large amount of nitrate that is cycled into stage A to enter denitrification and is eventually removed in the form of dinitrogen gas. In the A-tank, the anaerobic oxidation of ammonium may take place, which converts NH_4_^+^ into NO_3_^−^, NO_2_^−^, and, further, nitrogen gas [19].

### 3.5. Effect of Varying the Hydraulic Load on the Efficiency of Sewage Treatment

Next, the effect of the hydraulic load was investigated, as this can influence the regeneration speed of biofilms in the system, as well as the microbial activity of the microorganisms present and their residence time [20]. The hydraulic load was therefore initially set at 120 cm^3^ (cm^2^·d)^−1^ for 10 days, then decreased to 80 cm^3^(cm^2^·d)^−1^ for 10 days, to be reduced further to 60 cm^3^ (cm^2^·d)^−1^ for the last 10 days.

**Figure 6:**
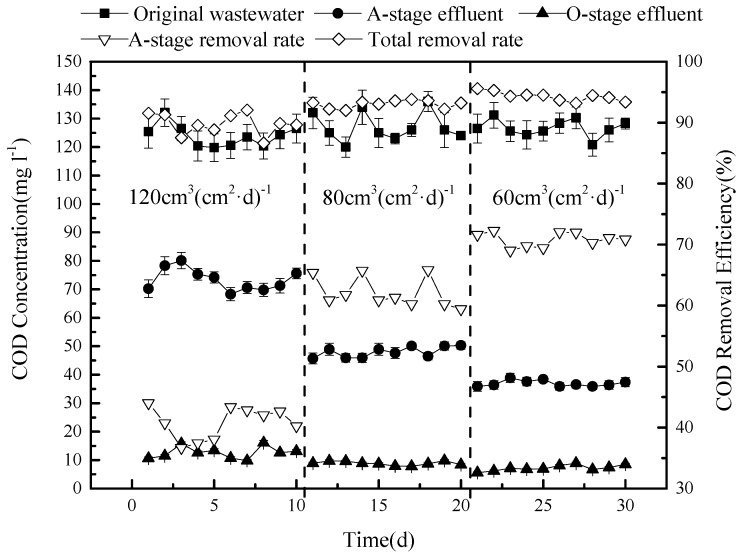
Variation in COD concentration (**top**) and COD removal efficiency (**bottom**) under three different hydraulic loads, with a decreased flow over time (120, 80 and 60 cm^3^ (cm^2^·d)^−1^) and a constant circulation capacity.

The experimental results (Figure 6) show that the overall average removal rate of COD increases from 89.8% to 94.3% as the hydraulic load is decreased. Under the tested conditions, the reflux rate was kept constant at 60 cm^3^ (cm^2^·d)^−1^, giving a circulation ratio between 50% for the highest tested load at the beginning of the experiment and 100% for the lowest tested load at the end of the experiment.

The results on the COD concentration in tank A are highly dependent on the hydraulic load; reducing the load results in a lower COD concentration in tank A, as measured at the outlet. It has only a marginal effect during the O-stage, although, for the whole system, decreasing the load slightly improves COD removal. This can be explained as follows. With a decreased hydraulic load, the flushing activity that may (partially) remove biofilms would be lower, which may favor COD removal. In addition, a lower load results in a larger circulation ratio, causing a stronger dilution effect at stage A. Moreover, the reflux solution is rich in nitrate, so a higher reflux ratio (at lower loads) will send more nitrate back into the A-stage. As a result, microorganisms will consume more carbon for denitrification at a lower load.

**Figure 7:**
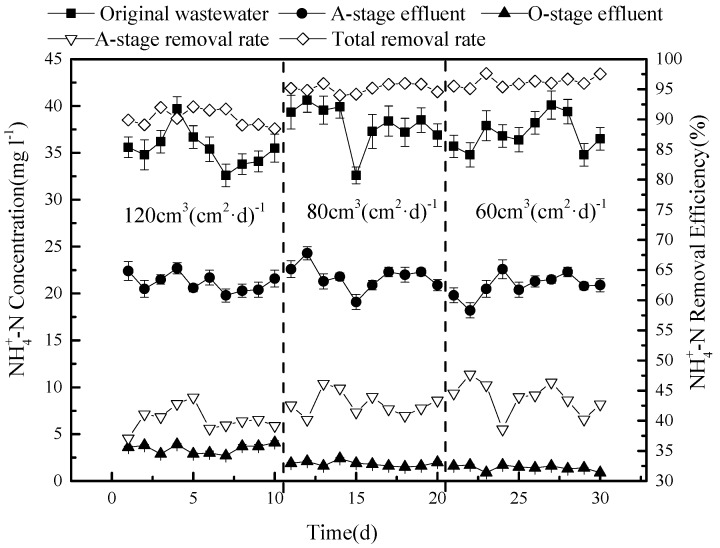
Variation in NH_4_^+^-N concentration (**top**) and removal efficiency (**bottom**) under the three different hydraulic loads.

The removal of NH_4_^+^-N was less dependent on the hydraulic load, as shown in Figure 7.

No difference in concentration was observed in tank A and only a small decrease in ammonium concentration was found in system O under a load of 120 and 80 cm^3^ (cm^2^·d)^−1^, respectively. As a result, the overall removal efficiency of NH_4_^+^-N slightly increased from 90.3 to 96.2%. 

**Figure 8:**
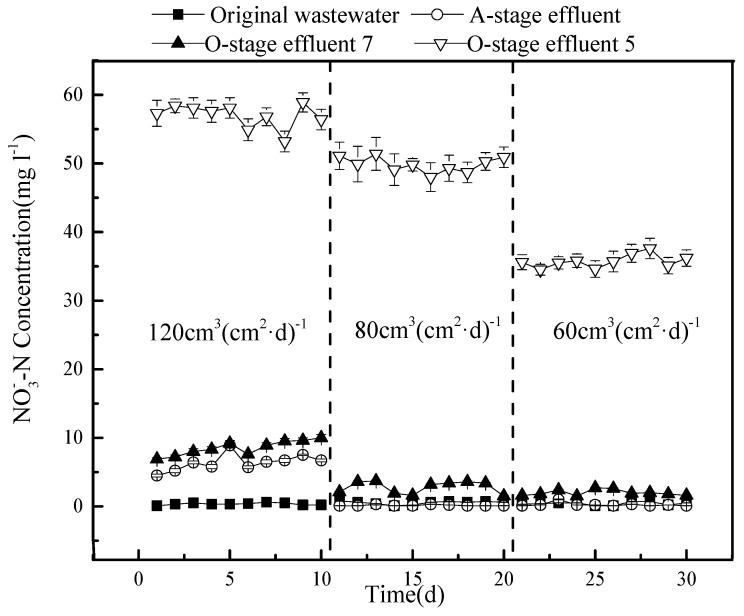
Variation in nitrate concentration under the three different hydraulic loads.

A much stronger effect is observed for nitrate removal (Figure 8).

The effluent from outlet 5 of the O-stage provides the values of the circulation fluid. Here, the nitrate concentration strongly decreases under a lower hydraulic load, with a larger drop when the flow is reduced from 80 to 60 cm^3^(cm^2^·d)^−1^. In contrast, changes in the A-stage and outlet 7 of the O-stage system are minor and may reflect dilution effects as the circulation ratio increases. The reasons for these observations may be the following: The higher the hydraulic load, the greater the amount of nitrate that enters the A-stage, and the less the degradation rate of denitrifying bacteria, which results in an accumulation of nitrate and higher concentrations in the effluent. A higher hydraulic load also shortens the hydraulic retention time in the A-stage system, allowing for less nitrate to be converted into nitrogen gas, which would escape. In addition, flushing out more bacteria under a higher hydraulic load may negatively affect the denitrification capacity of the A-stage.

**Figure 9:**
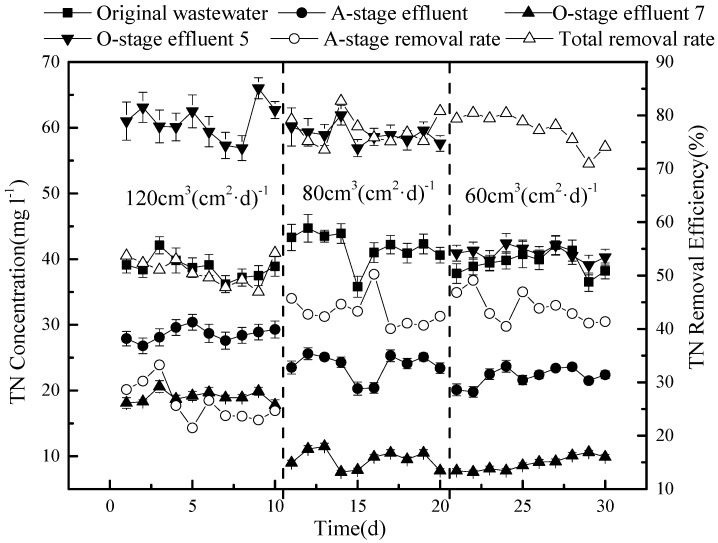
Variation in total nitrogen concentration under the three different hydraulic loads.

The effects observed for TN are a combination of the nitrate and ammonium results (Figure 9).

Overall, the removal efficiency of TN was low (only 50.8%) under the highest tested hydraulic load (120 cm^3^ (cm^2^·d)^−1^ with 50% circulation), but there was little difference between a load of 80 or 60 cm^3^ (cm^2^·d)^−1^, resulting in concentrations of 9.5 mg L^−1^ and 8.9 mg L^−1^ TN on average, corresponding to a removal efficiency of 77.4 and 77.2%, respectively. Based on the tested conditions, we conclude that a hydraulic load of 80 cm^3^ (cm^2^·d)^−1^ and a reflux ratio of 75% produces an optimal removal of both COD and nitrogen from sewage in the tested model.

### 3.6. Comparative Analysis of the Pollutant Removal Mechanism between the Traditional CSRI System and the CSRI System Combined with Pre-Denitrification

In the CSRI, the sewage flows through the filter material from the top down. To treat wastewater, percolation media and microorganisms on the medium absorb, intercept, and decompose pollutants in the water. The unique structure of CSRI, and its water intake mode of wet–dry cycling, make the microorganism phase on the surface of the percolation media very rich. The percolation medium has aerobic, aerobic-aerobic, and anaerobic functions so CSRI has a good wastewater treatment effect. The removal rate of COD_cr_ is between 85–90%, ammonia nitrogen is above 90%, and SS and LAS are above 95%. With a simple process and low investment, CSRI has obvious advantages and an important application value. However, CSRI’s removal efficiency of TN is poor at only 10–30%. CSRI has a higher removal rate of ammonia nitrogen, but the nitrate nitrogen content in the outlet water is increasing, so the removal efficiency of TN is poor. This shows that the denitrification of CSRI is weak. The main reasons are as follows: (1) In the CSRI system, ammonia nitrogen is mainly converted into nitrate nitrogen in the top of the filter. There are sufficient organic carbon sources on the top, although it is dominated by the aerobic environment, so the denitrification is inhibited; (2) the removal rate of organics in the upper part of the filter is high. The lower part of the filter is in an anaerobic environment for a long time, although it has a low nitrate content and lacks sufficient organic carbon source for denitrification. The denitrification process is inhibited. That is to say, there is a “dislocation” between the carbon source and oxygen required for nitrous nitrogen and denitrification; and (3) with a good permeability of CSRI, the nitrate nitrogen that migrates with the flow has a short residence time, and the denitrification time is short. The nitrate nitrogen in the water cannot be reduced to nitrogen to remove it.

In view of the advantages and disadvantages of the traditional CSRI system, this paper constructed an A/O two-stage pre-denitrification CSRI system. In this system, the nitrification liquid from the early-stage outflow of the O-stage is reintroduced into the A-stage. It makes full use of the carbon source and anaerobic environment of the wastewater in the A-stage to solve the dislocation phenomenon of the nitrate nitrogen, carbon source, and anaerobic environment. In the later stage, the effluent flowing out of the A-stage is directly discharged. In this paper, the removal rate of COD and ammonia nitrogen are higher than 93%, and the removal rate of TN is 76.7%. The reason is as follows:

(1) The concentration of nitrate nitrogen in the nitrification solution is high, while the concentration of COD and ammonia nitrogen are low. The COD and ammonia nitrogen in the original wastewater are diluted by returning to the A-stage so as to reduce the concentration of COD and ammonia nitrogen in the outlet water.
(2) Interception and adsorption of ${\mathrm{NH}}_{4}^{+}$-N and COD by the filter: ${\mathrm{NH}}_{4}^{+}$-N is positively charged and is easily absorbed by negatively charged media and microorganisms. Then, the microorganisms transform ${\mathrm{NH}}_{4}^{+}$-N.
(3) The O-stage mainly consists of nitrification: Under aerobic conditions, nitrifying bacteria convert ammonia nitrogen into nitrate nitrogen. When the sewage passes through the O-stage, on the one hand, the ammonia nitrogen and COD in the sewage are absorbed by the filter material. On the other hand, the effluent scours the O-stage and dissolves the nitrate nitrogen. Due to the high solubility of nitrate nitrogen in the water, when the sewage contacts the filter, a large amount of nitrate nitrogen flows out with the sewage, resulting in a higher concentration of nitrate nitrogen in the nitrification solution of the earlier effluent system. At the same time, the total amount of nitrate nitrogen in the system is limited. Even though the sewage that enters the system later has a scouring effect on the filter, the sewage that enters in the earlier phase takes a large amount of nitrate nitrogen away as nitrification liquid back flow, so that the concentration of nitrate nitrogen is lower in the sewage directly discharged into the environment in the later phase, thus improving the denitrification efficiency.
(4) The nitrification liquid flows back into the A-stage, which is in a state of full water, providing an anoxic environment for denitrifying bacteria. The nitrification liquid provides nitrification nitrogen for nitrification bacteria, and the original wastewater provides a carbon source for the nitrification of bacteria. In this way, the denitrification of the system can be enhanced.

Compared with the processing of activated sludge by an anaerobic/oxic A/O process, the pre-denitrification rapid infiltration system has the greatest advantage in that it can concentrate nitrate nitrogen into a small amount of nitrification liquid and then send this back into the A-stage for denitrification. The traditional processing of activated sludge by an A/O process increases the system’s denitrification efficiency only by increasing the reflux ratio because the concentrations of all the pollutants in the aerobic pool are uniformly mixed. For the total nitrogen removal efficiency to be 75% (actually, less than 75%), the reflux ratio needs to be increased to 300%. The increase of the reflux ratio leads to an increase in the cost as well as the dissolved oxygen that enters the A-stage, which will have some negative effects on denitrification.

## 4. Conclusions

In this experiment, a CSRI system with a two-stage A/O process was combined with pre-denitrification and circulation, which fully combines the advantages of both aerobic and anoxic denitrification for wastewater treatment. By determining the removal rate of COD, NH_4_^+^-N, and TN, and testing different hydraulic loads with a constant amount of backflow nitrification, we determined the optimal conditions for sewage treatment. The obtained average removal rates of NH_4_^+^-N and COD were 95.4% and 96%, respectively, under a hydraulic load of 80 cm^3^ (cm^2^·d)^−1^ and a circulation ratio of 75%. The average removal rate of TN was 77.4% under these conditions, which is much higher than that of conventional CSRI systems, without reducing the removal rate of NH_4_^+^-N and COD. The experimental principle is simple and easy to implement, so the practical application of this system is highly likely. Provided the observations can be maintained after scaling up, the solution solves the problem of traditional CSRI systems, as we have provided a theoretical basis for the pretreatment of denitrification in a CSRI system to treat domestic sewage.

## Figures and Tables

**Table 1:** The initial concentration of the chemical oxygen demand (COD), ammonia (NH_4_^+^-N), nitrate nitrogen (NO_3_^−^–N), total nitrogen (TN), pH, and temperature (T) in wastewater.

Characteristics	COD (mg/L)	NH_4_^+^-N (mg/L)	NO_3_-N (mg/L)	TN (mg/L)	pH
MEAN	129.6	36.9	0.7	40.4	6.72
SD	10.1	2.5	0.2	1.4	0.04

## Abbreviations

A/O	anaerobic-oxic process
COD	chemical oxygen demand
CSRI	Constructed Soil Rapid Infiltration system
TN	total nitrogen
T	temperature
